# Effect of high-speed sintering on the marginal and internal fit of CAD/CAM-fabricated monolithic zirconia crowns

**DOI:** 10.1038/s41598-023-44587-5

**Published:** 2023-10-11

**Authors:** Seulgi Lee, Gyujin Choi, Jinhyeok Choi, Youngjun Kim, Hee-Kyung Kim

**Affiliations:** 1 Department of Dental Public Health, Graduate School of Clinical Dentistry, Ajou University, Suwon, Republic of Korea; 2 Imagoworks Inc., Seoul, Republic of Korea; 3 Department of Prosthodontics, Institute of Oral Health Science, Ajou University School of Medicine, Suwon, Republic of Korea

**Keywords:** Engineering, Materials science, Mathematics and computing

## Abstract

This study compared the marginal and internal fit of zirconia crowns fabricated using conventional and high-speed induction sintering. A typodont mandibular right first molar was prepared and 60 zirconia crowns were fabricated: 30 crowns using conventional sintering and 30 crowns using high-speed sintering. We presented a new evaluation methodology to measure the marginal and internal fit of restorations through digital scanning, aligning the two datasets, and measuring the distance between two arbitrary point sets of the datasets. For the marginal fit, we calculated the maximum values of the shortest distances between the marginal line of the prepared tooth and that of the crown. The calculated values ranged from 359 to 444 μm, with smaller values for the high-speed sintered crowns (*P* < 0.05). For the internal fit, we employed mesh sampling and computed the geodesic distances between the prepared tooth surface and the crown intaglio surface. The measured values ranged from 177 to 229 μm with smaller values for the high-speed sintered crowns, but no significant difference was found (*P* > 0.05). Based on our results, the high-speed sintering method can be considered a promising option for single-visit zirconia treatment in dental practice.

## Introduction

Yttria-stabilized tetragonal zirconia polycrystal has been widely used in dentistry as a promising biomaterial owing to its biocompatibility, excellent mechanical properties, and high chemical stability. In particular, full-contour monolithic zirconia restorations have gained increasing popularity in terms of reduced tooth reduction and minimal wear of antagonist teeth^1^. With the advancement of digital technologies in dentistry, the fabrication of monolithic zirconia restorations involves the use of computer-aided design (CAD) and computer-aided manufacturing (CAM), which eliminate the need for manual fabrication. CAD/CAM production offers more precise and accurate dental prostheses than traditional methods that are vulnerable to human error.

Traditional dental practice often involves a multi-visit process to complete a dental restoration, and patients often have to wear uncomfortable temporary crowns between visits. However, it is now possible to complete certain types of ceramic restorations in a single visit with advancements in CAD/CAM technology, thereby saving time for both dentists and patients^2^. While single-visit ceramic restorations are becoming increasingly common, zirconia restorations typically require an 8–12 h sintering process, as part of their fabrication. Pre-milled zirconia is heated to a high temperature varying between 1350 and 1550 °C for the consolidation and densification of zirconia particles, leading to a significant increase in strength, durability, and aesthetics^3^.

In conventional sintering processes, the heating elements are responsible for converting electrical energy into heat energy by controlling the heating rate up to 70 °C/min to ensure uniform heating throughout the furnace. Heating elements are generally made of silicon carbide (SiC) or molybdenum disilicide (MoSi_2_) owing to their high-temperature resistance and excellent thermal conductivity^4^. SiC elements are considered cost-effective and can be used at temperatures up to 1600 °C^4^. MoSi_2_ elements have a longer lifespan than SiC elements and can withstand even higher temperatures (up to 1800 °C), making them suitable for applications requiring extremely high temperatures^5^. The choice between SiC and MoSi_2_ heating elements depends on the specific requirements of the application, including temperature range, durability, and budget considerations.

Microwave sintering is regarded as a relatively new processing technique for zirconia materials, differing from conventional sintering by utilizing electromagnetic radiation to achieve high temperatures. Particularly, microwave sintering can provide a reduced processing time (2–4 h) and energy consumption, exhibiting similar fracture toughness^6^, flexural strength^7^, and translucency^8^ of dental zirconia compared to those with conventional sintering. However, several aspects still need to be explored in terms of productivity, as microwaves can only penetrate a short distance in materials and typically sinter only a few dental restorations at a time^9^.

High-frequency induction heated sintering (HFIS) is a novel, rapid sintering method developed for the fabrication of ceramics and composites. In HFIS, a high-frequency alternating current passes through an induction coil to generate a rapidly changing electromagnetic field, resulting in accelerated densification and reduced processing times^10^. Induced eddy currents within conductive components, such as metal powders or carbon fibers, generate resistive heating and a localized temperature rise^11^. The recently introduced CEREC SpeedFire sintering furnace (Dentsply Sirona, Charlotte, USA) can sinter a monolithic zirconia crown in 10–15 min using induction technology^12^. According to the manufacturer’s instructions, the CEREC SpeedFire reaches a maximum heating rate of 300 °C/min and there is no need to preheat, maintain a holding temperature, or undergo a drying process, which takes several hours. With this technology, the entire process of obtaining a zirconia crown can be completed in a single office visit, thereby saving time and reducing the need for temporary restorations. However, various technical and clinical investigations must be conducted to ensure its reliability, safety, and efficacy bacause the sintering process is the final step in which the material is hardened and its properties are optimized. Some studies stated that high-speed sintering resulted in similar^13^ or even higher^14^ mechanical properties of zirconia crowns compared to conventional methods, while others argued that high-speed sintering impaired their translucency and mechanical properties^12,15^.

From a clinical perspective, a precise fit of zirconia restorations is essential for maintaining periodontal health, preventing cement dissolution, and ensuring optimal retention and stability of the prosthesis^16^. Absolute marginal discrepancy, which is defined as “the angular combination of the marginal gap and extension error”^17^, considers both horizontal and vertical directions confirming that the components are correctly positioned and aligned. This can reflect the overall discrepancy between the prepared tooth structure and the restoration’s margin^18^. Internal misfit refers to how well the internal surface of the restoration conforms to the contours of the tooth preparation^19^. A precise internal adaptation reduces the risk of microleakage and promotes even distribution of occlusal forces, thereby reducing the risk of fracture^19,20^. Several techniques have been used to assess the misfit of dental restorations. The cross-sectional method^21^ and replica technique^22^ are two common approaches for directly measuring the cement film thickness beneath restorations. Micro-computed tomography (CT) is an accurate and reliable method that provides volumetric information nondestructively^23^. However, the resolution of micro-CT can be influenced by various factors, including the size of the X-ray detector pixels and focal spot size^24^. Recently, digital techniques have been increasingly employed to evaluate marginal discrepancies in dental restorations, providing advantages over traditional measurement methods in terms of precision, repeatability, efficiency, and data sharing^25,26^.

The fit of zirconia restorations sintered in a high-speed induction furnace has been evaluated in a few studies, but with limited reliability. Elisa Kauling et al.^27^ reported smaller marginal gaps in 3-unit zirconia restorations with high-speed sintering compared to those with conventional sintering, by using the replica technique. Antón et al.^28^ suggested that the high-speed sintering process may have disadvantages in terms of marginal fit, depending on the type of restorations. They also described possible inaccuracies in the positioning of the abutment teeth during measurements. Therefore, this study aims to compare the marginal and internal fit of zirconia crowns fabricated using conventional and high-speed sintering methods, employing an innovative investigation method to minimize measurement errors. The comparison was made through a digital workflow that involved the following steps: digital scanning, segmentation of the region of interest (ROI), reference best-fit alignment, Hausdorff distance measurement, and geodesic distance measurement between the surface meshes. The null hypothesis tested was that there would be no difference in the marginal and internal fit of monolithic zirconia crowns using conventional and high-speed sintering methods.

## Methods

### Sample preparation

**Figure 1:**
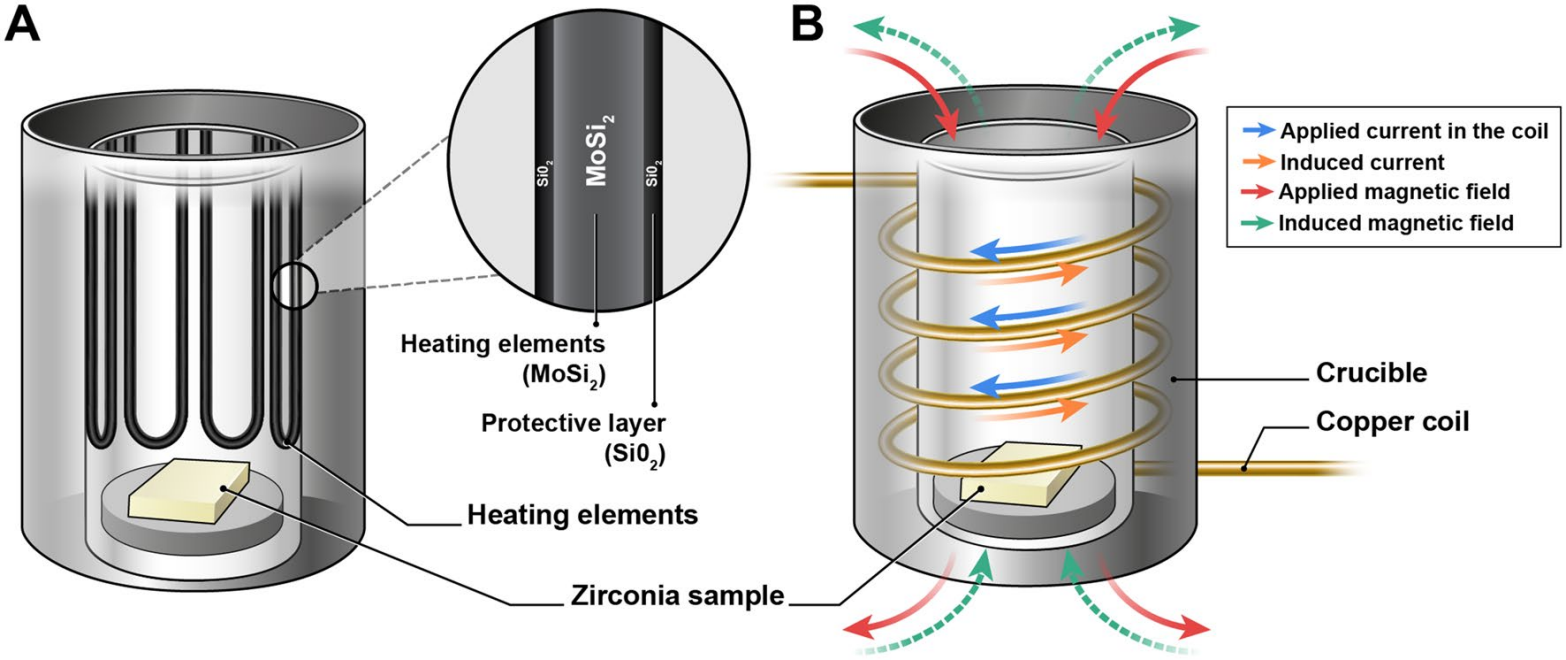
Schematic diagrams of the sintering furnaces. (**A**) A conventional sintering furnace with MoSi_2_ heating elements which convert electric energy into heat through the Joule heating process. (**B**) By using electromagnetic heat, or induction, a high-speed sintering furnace requires short interaction times to reach the required temperature.

**Figure 2:**
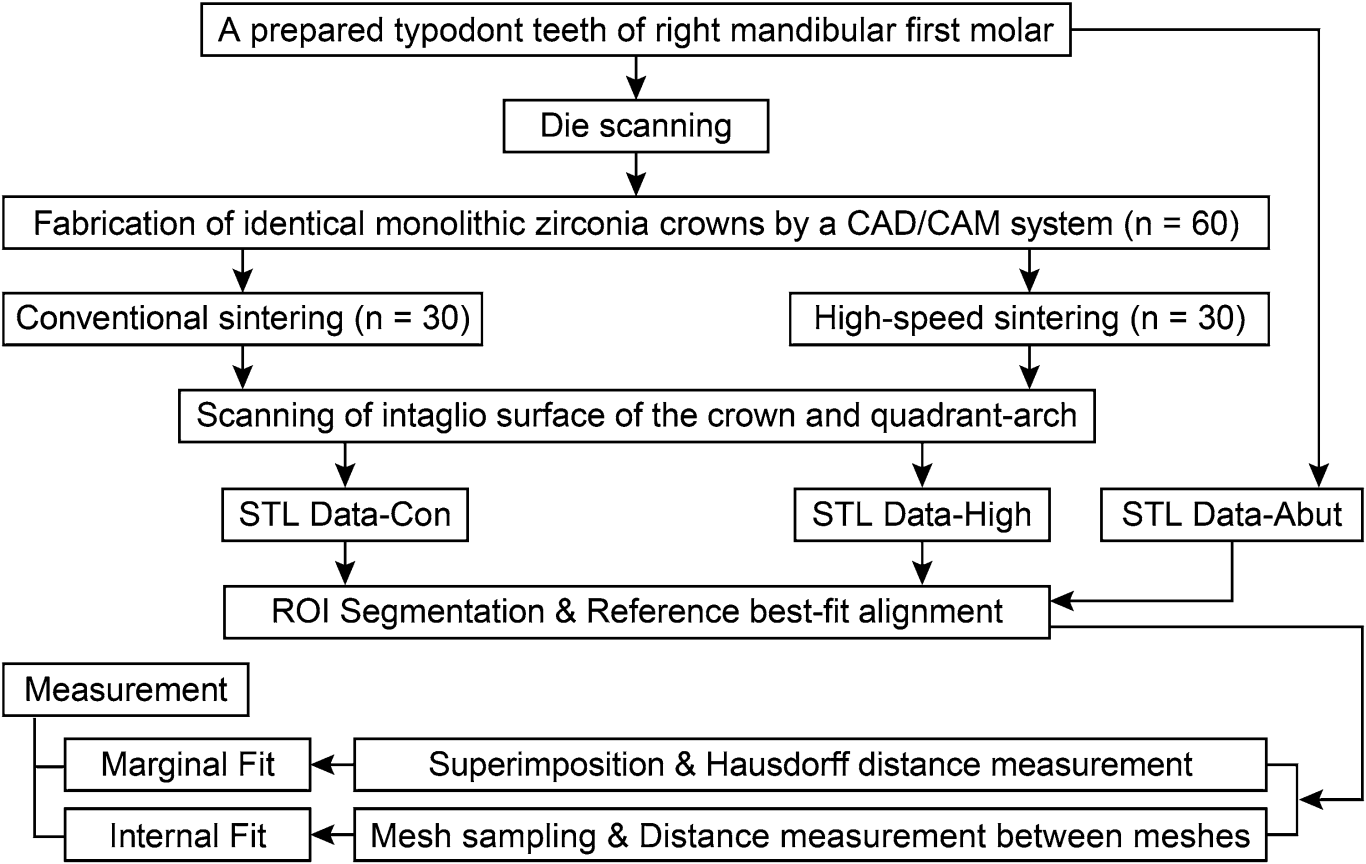
Schematic diagram of the study design. STL Data-Con, the standard tessellation language (STL) dataset format obtained from conventionally sintered crowns. STL Data-High, the STL dataset format obtained from high-speed sintered crowns. STL Data-Abut, the STL data format obtained from the prepared typodont tooth. *ROI* region of interest.

A typodont mandibular right first molar was prepared with axial reduction of 1.2 mm, occlusal reduction of 1.0 mm, and chamfer margin of 1.0 mm width. The quadrant-arch typodont model containing the prepared tooth was scanned using an intra-oral scanner (CEREC Primescan; Dentsply Sirona, Charlotte, NC, USA) to obtain 3D scan mesh files. A full-contour monolithic crown was designed virtually by using a dental CAD software (Dental Designer; 3Shape, Copenhagen, Denmark) with a cementation spacer (defined as the digital spacer thickness in the software) of 40 μm on the occlusal and axial surfaces of the prepared tooth, starting 0.5 mm above the finish lines of the tooth. Sixty identical crowns were milled from partially sintered zirconia blanks (KATANA ML; Kuraray Noritake Dental, Osaka, Japan) and divided, according to the sintering methods, into two groups: conventional and high-speed sintering (n = 30/group). The Tiger-S (GMD BIO, Seoul, Korea) was used for the conventional sintering group, and the CEREC SpeedFire (Dentsply Sirona, Charlotte, USA) was used for the high-speed sintering group. Conventional sintering was performed at a heating rate of 10 °C/min and a dwell time of 120 min at a final temperature of 1500 °C, whereas high-speed sintering was conducted at a heating rate of 300 °C/min and a dwell time of 2 min at 1500 °C. High-speed sintering required only 15 min to complete, whereas conventional sintering required approximately 8 h. Detailed schematics of the furnaces are shown in Fig. 1. An overview of the study design is depicted in Fig. 2.

### Alignment and marginal fit measurements

**Figure 3:**
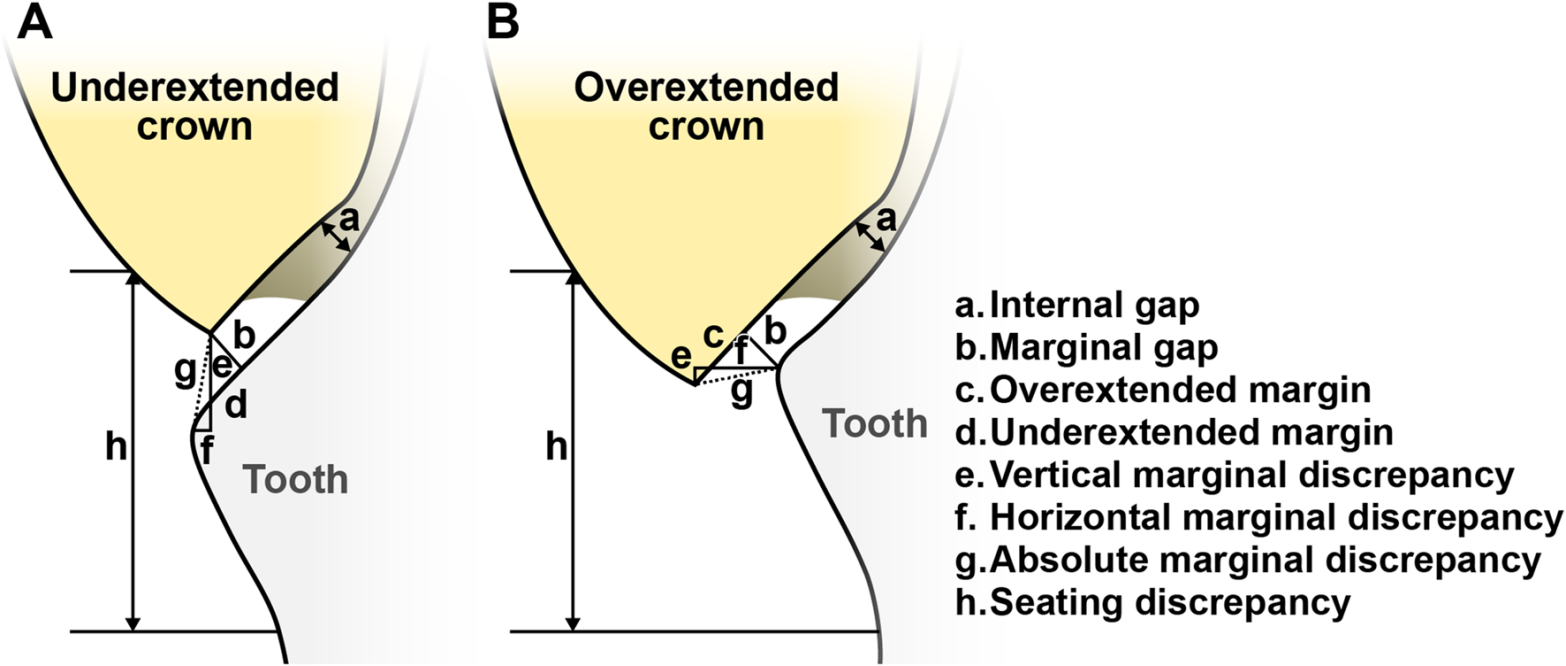
Terminology of dental restoration misfit for (**A**) underextended and (**B**) overextended crowns^17^.

**Figure 4:**
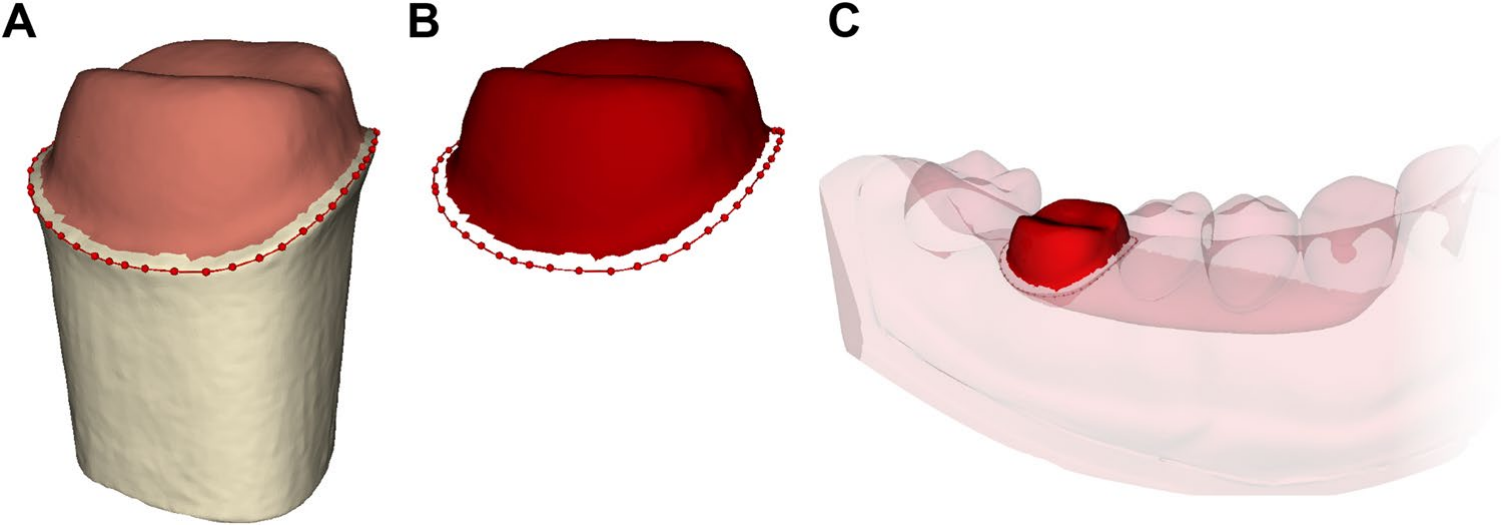
(**A**) Point annotation of 50 marginal line landmarks. (**B**) Determination and extraction of regions of interest (ROIs). (**C**) Alignment of ROIs to the reference image.

We measured the absolute marginal discrepancy as an indicator of marginal fit (Fig. 3). A quadrant-arch mesh model was used as the alignment reference. The prepared tooth was then removed from the typodont and scanned. The marginal line on the prepared tooth die, which is defined as the boundary curve of the contact area between an abutment and a restoration^29^, was manually annotated using a software (3D Slicer, Version 4.10.2; Surgical Planning Laboratory, Boston, MA, USA). This involved point annotation of 50 margin landmarks on the abutment by a single experienced investigator (S.L.) (Fig. 4A). The ROIs, including the marginal line and abutment surface 0.5 mm above the marginal line (Fig. 4A and B), were extracted and subsequently aligned (Fig. 4C) to the reference mesh model using an artificial-intelligence-based alignment program (Dentbird Solutions; Imagoworks, Seoul, Korea).

**Figure 5:**
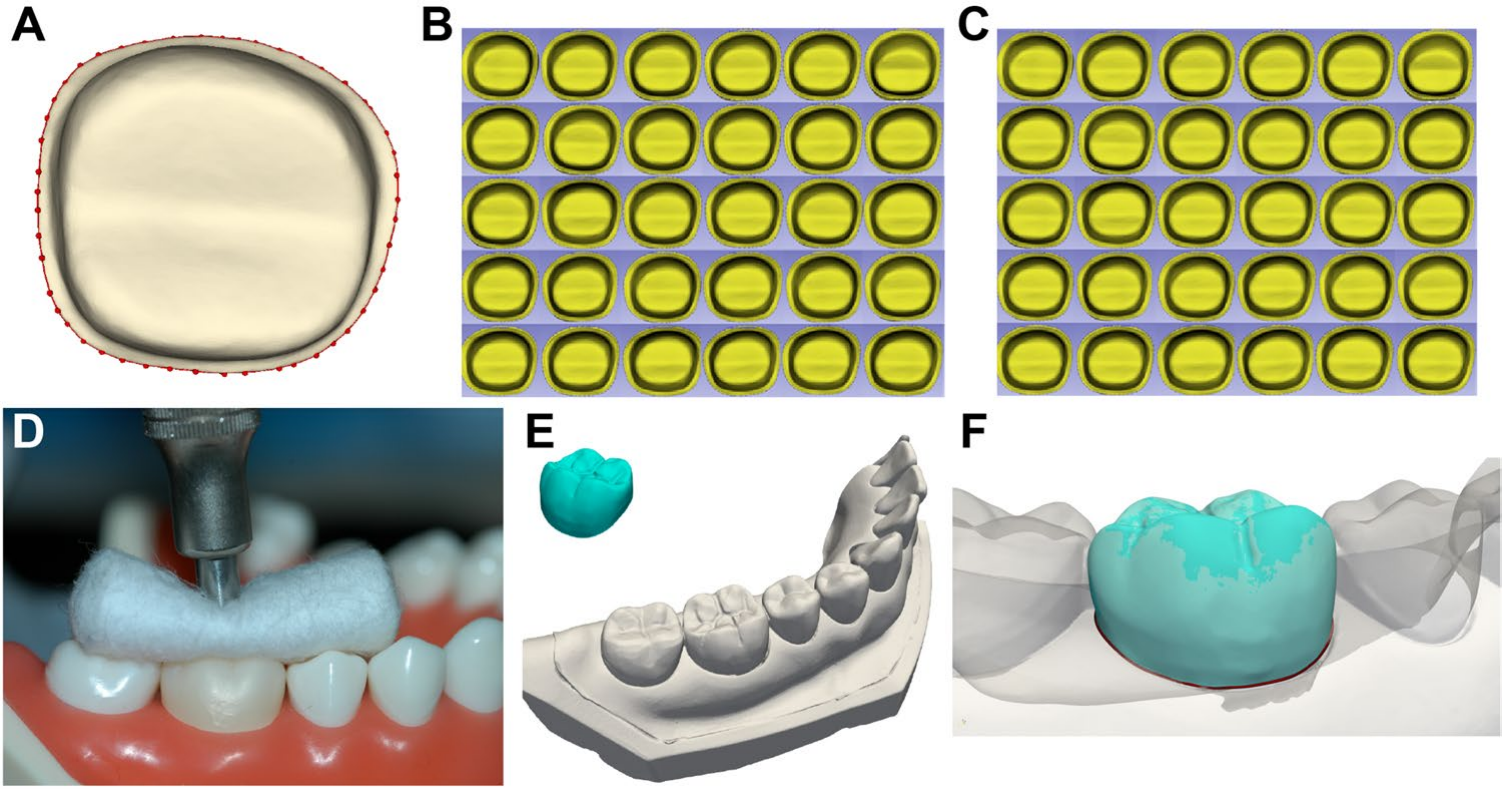
(**A**) Point annotation of 50 margin landmarks on the restoration. Point annotation on the 30 crowns with a conventional sintering protocol, (**B**) and 30 crowns with a high-speed sintering protocol (**C**). (**D**) Each crown was seated on the prepared typodont tooth under a constant load of 50 N. (**E**) and (**F**) Each crown superimposed on the reference mesh model.

The intaglio surfaces of all zirconia crowns were scanned using an intra-oral scanner (CEREC Primescan; Dentsply Sirona, Charlotte, NC, USA). For each crown, a single experienced investigator (S.L.) performed point annotation of 50 margin landmarks on the restoration (Fig. 5A–C). Each crown was then seated on the typodont model under a constant load of 50 N using a cementation device (Fig. 5D) and the quadrant-arch containing each crown was scanned again as the reference data. No cement was used for fixation. Subsequently, each crown was superimposed on the reference mesh model using the same alignment workflow as that used for the abutment (Fig. 5E and F).

**Figure 6:**
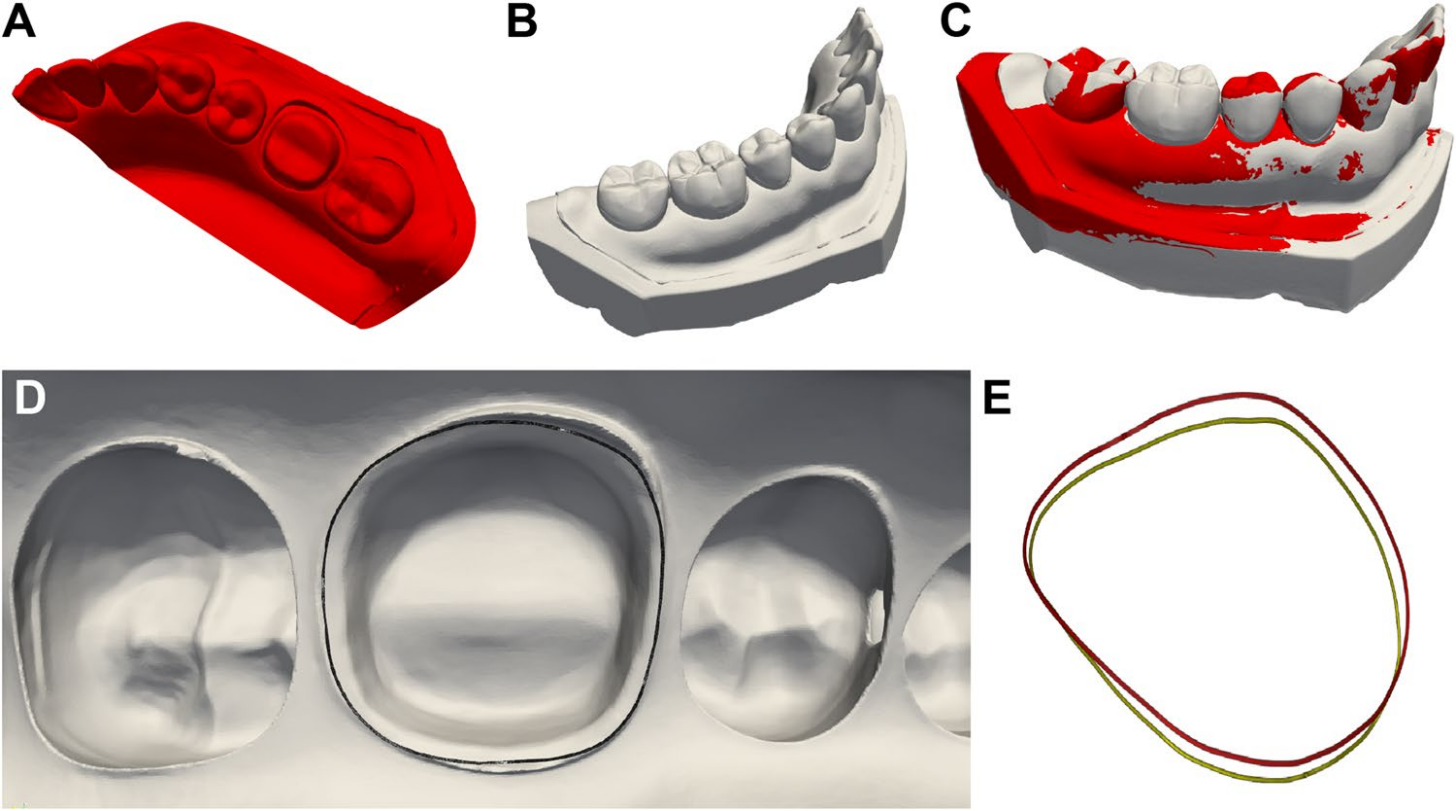
The quadrant-arch mesh model with a prepared tooth, (**A**) was superimposed onto the model with each crown (**B** and **C**). (**D**) An internal view of the aligned marginal lines of the mesh models. (**E**) Extracted marginal lines compared using Hausdorff distance: the green and red lines indicate the marginal line of an abutment and a restoration, repectively.

The quadrant-arch mesh model with a prepared tooth was superimposed onto the model with each crown of the conventional-sintering group, and the marginal lines were compared using the Hausdorff distance with a software (Dentbird Solutions; Imagoworks, Seoul, Korea) (Fig. 6). The Hausdorff distance is the maximum distance between any point in the first set and its nearest point in the second set, and vice-versa^30^. For the high-speed sintering group, the marginal discrepancies were measured using the same method. The means of the Hausdorff distances of crowns fabricated using conventional and high-speed sintering methods were compared.

### Mesh sampling and internal fit measurements

**Figure 7:**
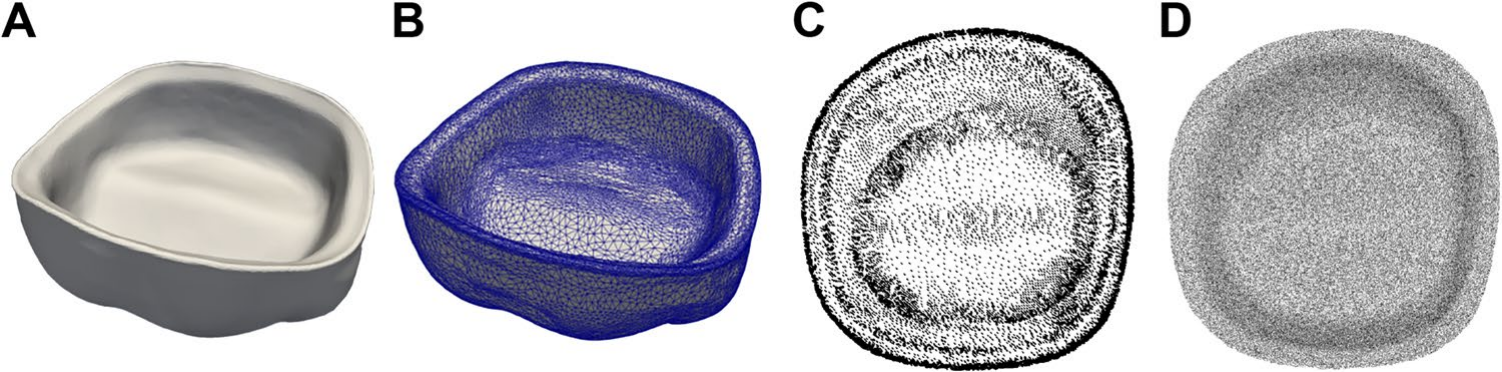
The surface-based data, (**A**) was transformed into a three-dimensional mesh file, (**B**) to generate the node and element data structures. Density map before, (**C**) and after, (**D**) mesh sampling.

To determine the internal fit of the zirconia crowns in each sintering group, the distance between the prepared tooth surface and the crown intaglio surface was calculated using point cloud analysis. Mesh sampling and optimization processes were conducted using a triangular mesh to achieve an even distribution of points on the surface using a software (Dentbird Solutions; Imagoworks, Seoul, Korea) (Fig. 7). A dart-throwing algorithm was used to generate a Poisson disk-point set^31^.

With a uniform mesh density, geodesic distances (shortest paths) were calculated between any pair of mesh nodes, which contained a mesh node on the tooth surface and a mesh node on the crown intaglio surface. These distances were then used as a measure of dissimilarity and were described using histograms. The mean distances between the zirconia crowns fabricated using the conventional and high-speed sintering methods were compared.

### Statistics

All statistical analyses were conducted using a software (IBM SPSS Statistics for Windows, v25.0, IBM Corp., Chicago, IL, USA) with a significance level of α = 0.05. An independent-sample *t* test was used to determine the significance of the difference in the mean values between the two sintering groups.

## Results

### Comparison of marginal fit

The Shapiro‒Wilk test indicated that the variables were normally distributed (*P* > 0.05). The maximum absolute marginal discrepancy was evaluated by measuring the Hausdorff distance between the marginal line of the prepared tooth and that of the crown. The results of the independent-sample *t* tests are presented in Table 1. The maximum absolute marginal discrepancies of the conventionally sintered crowns and those of the high-speed sintered crowns were significantly different; t(58) = – 2.150, *P* = 0.036. These results suggest that the sintering method affected the maximum absolute marginal discrepancies of the zirconia crowns. Specifically, our results suggest that when zirconia crowns are sintered in a high-speed furnace, the marginal discrepancy decreases.

**Table 1:** Comparison of the maximum values of the absolute marginal discrepancy between the crowns fabricated using conventional and high-speed sintering methods.

Group	Maximum value of absolute marginal discrepancy		
	Mean (μm)	SD	t (*P*)
Conventional sintering	419.384	24.558	– 2.150 (0.036)*
High-speed sintering	400.482	41.430	

*Significance determined by independent-samples *t* test, *P* < 0.05.

### Comparison of internal fit

The internal fit was evaluated by calculating the geodesic distances between two mesh surfaces: the prepared tooth and the crown intaglio surfaces. The results of the independent-sample *t* tests are shown in Table 2. The internal fit of the crowns fabricated with conventional and high-speed sintering were not significantly different, although a smaller mean value was found in the high-speed sintering group; t(58) = – 1.019,* P* = 0.313. These results suggest that the new high-speed sintering method does not change in the internal fit of zirconia crowns compared with that of conventionally sintered crowns.

**Table 2:** Comparison of the internal fit between the crowns fabricated using conventional and high-speed sintering methods.

Group	Internal fit		
	Mean (μm)	SD	t(*P*)
Conventional sintering	200.426	28.6108	– 1.019 (0.312)
High-speed sintering	194.170	17.6469	

**Figure 8:**
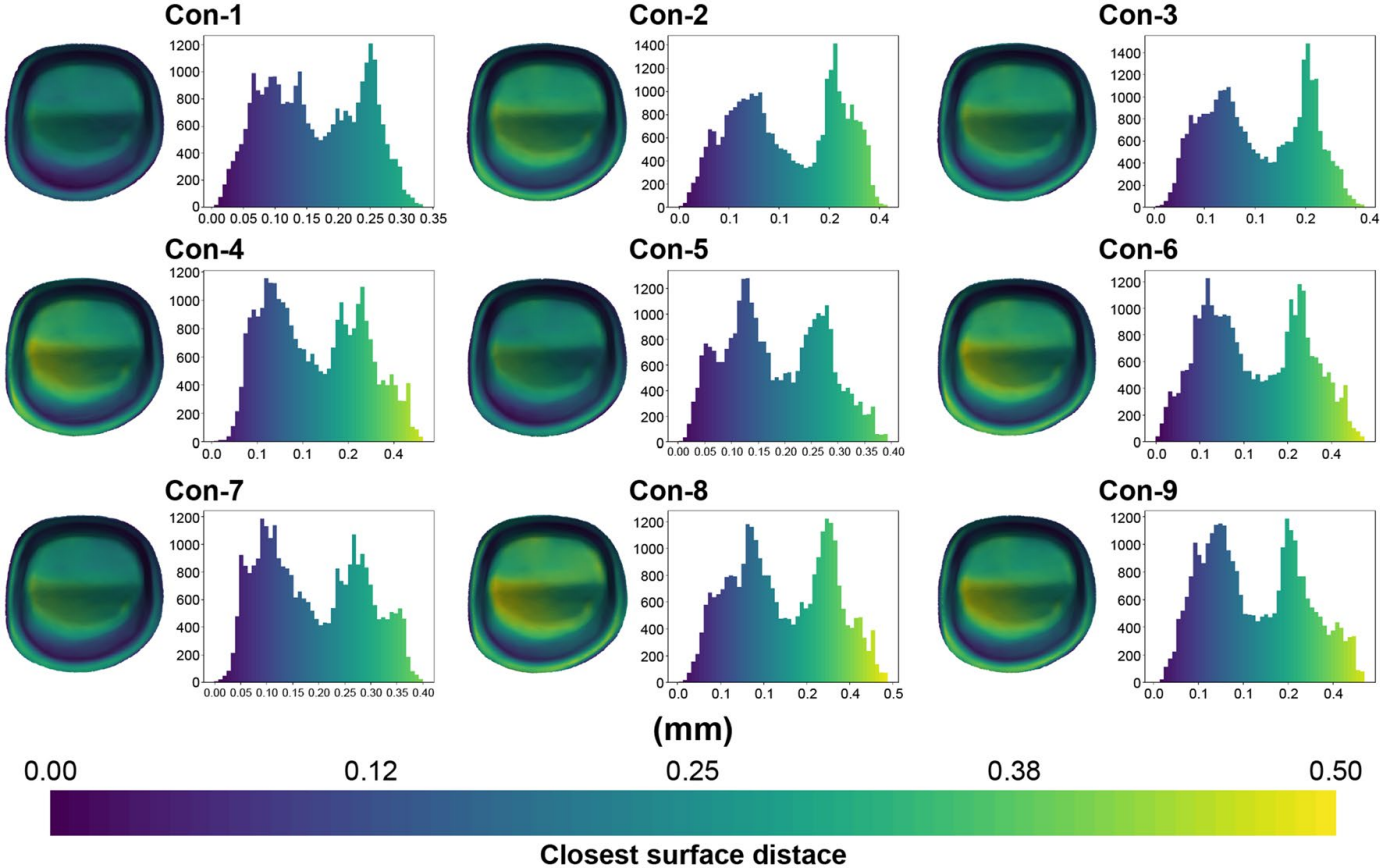
Representative color-difference maps of internal discrepancy measurements for the conventional sintering group.

**Figure 9:**
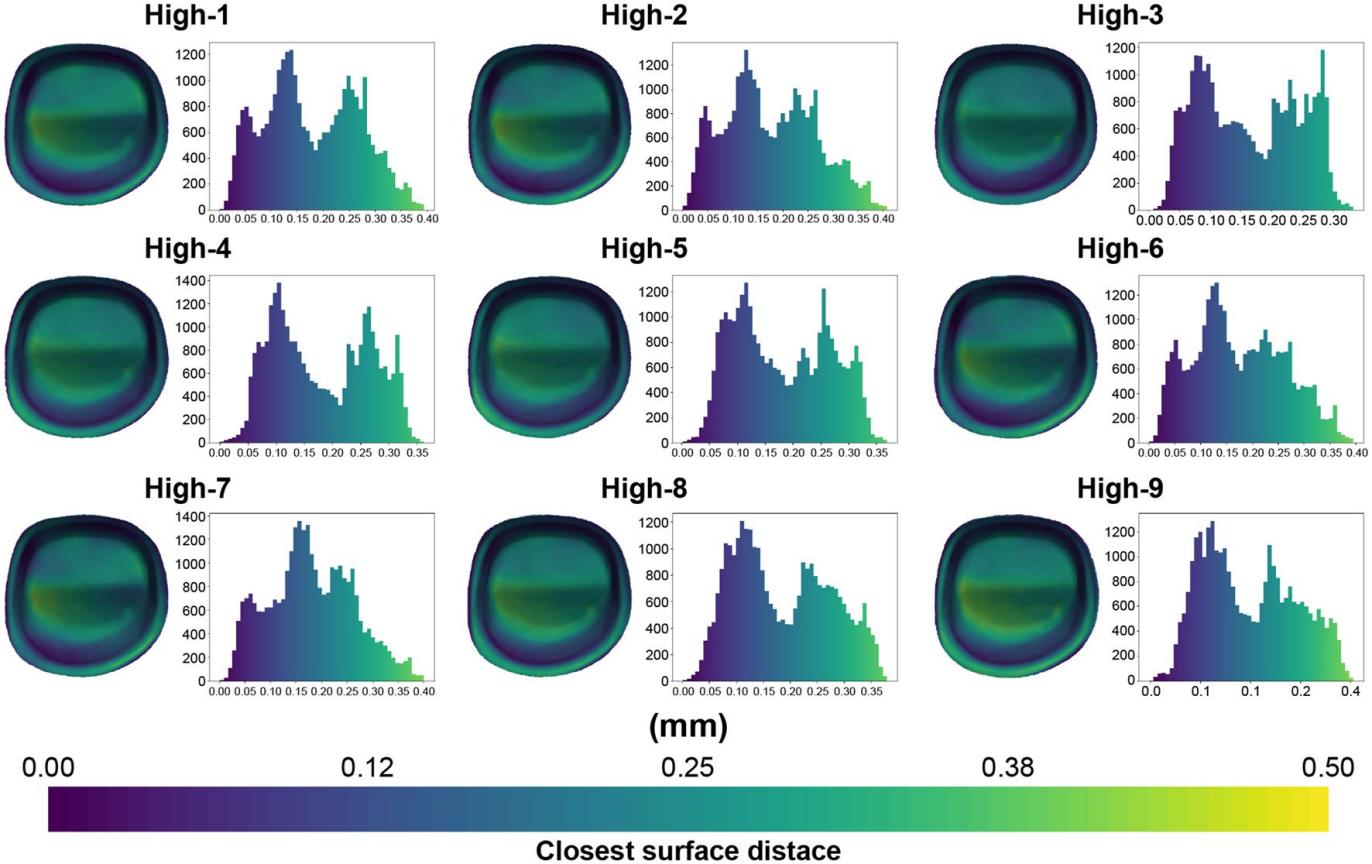
Representative color-difference maps of internal discrepancy measurements for the high-speed sintering group.

Figures 8 and 9 show the representative color-difference maps and histograms depicting the geodesic distances between the tooth and crown surfaces using the conventional and high-speed sintering methods, repectively. The internal fit discrepancies between the two sintering groups were not evenly distributed, with the highest discrepancies in the occlusal regions.

## Discussion

This study compared the marginal and internal fit of zirconia crowns fabricated using conventional and high-speed sintering methods. Regarding the marginal fit, the null hypothesis was rejected, as crowns sintered using the high-speed method demonstrated a superior marginal fit compared to those sintered using the conventional method. In contrast, in terms of the internal fit, the null hypothesis was accepted, as there was no significant difference in the internal fit values between the crowns fabricated using different methods.

Numerous studies have evaluated the clinically acceptable marginal and internal gaps in dental restorations. Although values may vary depending on factors such as restoration design, materials, impression techniques, fabrication methods, and cement types, many previous studies have considered ranges of 100–250 μm and 100–200 μm as clinically acceptable for the marginal and internal fit, respectively^18,20,32–36^. However, some studies did not specify whether they measured the horizontal or absolute marginal gap. Additionally, most studies measured the mean values of the marginal opening. With regard to internal fit, many studies have conducted two-dimensional area measurements by selecting 10–40 sites on the cross-sections for evaluation^34–36^.

In this study, we employed a state-of-the-art algorithm to compare the marginal and internal fit of crowns fabricated using different methods. To minimize manufacturing errors, all crowns were measured on a single reference die without the use of cement, and a single type of scanner was used. To measure the dissimilarity between the two images, we compared two-point sets consisting of a finite number of points by considering the 3D spatial position of each individual point. We assessed the misfit of zirconia crowns using the term 'absolute marginal discrepancy’ to refer to the measurement of the marginal fit. Instead of calculating the mean values of the marginal gap, we focused on calculating the maximum values of the shortest distance between 3D point sets. These maximum values indicate the maximum marginal deviation between the abutment and the crown. This approach constitutes a key aspect of the evaluation strategy. Hence, our values for marginal fit (359–444 μm) were higher compared to those in previous studies. In terms of the internal fit, we calculated the dimensional difference between the reference surface of the prepared tooth and the intaglio surface of the crowns using a mesh sampling technique and the computed geodesic distances on the meshes. Instead of conducting measurements at specific sites on the cross-sections as in previous studies, we computed an infinite number of the shortest paths of two arbitrary point sets on the mesh surface. The resulting values, 177–229 μm, were within the range of those found in previous studies.

In this study, we attempted to minimize the alignment errors in the investigated data sets by employing a reference best-fit alignment technique. Several studies have compared the alignment accuracy across different types of alignment methods, and have suggested that a reference best-fit alignment technique is considered superior to landmark-based alignment or best-fit alignment methods because it reduces alignment errors and improves repeatability^37,38^. This is because the reference best-fit alignment technique aligns two datasets based on changes experienced below a predefined threshold value.

The results of this study showed that zirconia crowns fabricated using the high-speed sintering process exhibited superior marginal and internal fit compared to those fabricated using the conventional sintering method, although there was no statistically significant difference in the internal fit of the crowns with both methods. These results may be attributed to the higher sintering shrinkage in the zirconia crowns fabricated using the conventional sintering method, leading to a greater misfit. In addition, we observed that the internal fit discrepancy was nonuniform, with larger gaps observed in the occlusal regions for both methods. However, a greater degree of distortion was observed in the conventionally sintered crowns. Therefore, the sintering method may affect the final shrinkage of the fabricated zirconia crowns. The limitation of this study is that a single brand of material and a single type of restoration with a fixed cementation spacer were used. Finally, more studies on the fit precision of speed-sintered zirconia restorations with varying materials, types of restorations, and cementation spacers are needed to verify the clinical reliability of the high-speed sintering method. Within the limitations of this study, in which specific types of conventional and high-speed furnaces were utilized, we can consider the high-speed sintering method as a promising option for single-visit zirconia treatment in dental practice.

## Conclusion

Within the limitations of this study, the sintering methods influenced the marginal and internal fit of zirconia crowns. As a new approach, we compared the marginal and internal fit between zirconia crowns fabricated with conventional and high-speed sintering methods through digital scanning, ROI segmentation, reference best-fit alignment of two images, mesh sampling, and distance measurements in metric spaces. Based on the results of this study, zirconia crowns subjected to high-speed sintering exhibited better marginal and internal fit than those subjected to conventional sintering. Therefore, high-speed induction sintering can be considered a valid option for single-visit dental treatments, especially concerning the marginal and internal gaps of fixed zirconia restorations.

## Data Availability

The datasets generated during the current study are available from the corresponding author (H.-K.K.) on reasonable request.
